# Value cocreation among spectators in professional spectator sports events: Scale development and effects of social media strategies

**DOI:** 10.1371/journal.pone.0320927

**Published:** 2025-05-23

**Authors:** Chen-Yueh Chen, Yi-Hsiu Lin, Che-Yi Yang, Ya-Lun Chou, Tzu-Yun Yeh

**Affiliations:** 1 Doctoral Program for Transnational Sport Management and Innovation, National Taiwan Sport University, Taoyuan, Taiwan; 2 Master Program of Sport Facility and Health Promotion, National Taiwan University, Taipei, Taiwan; 3 Department of Physical Education, Fu Jen Catholic University, New Taipei, Taiwan; Gelisim University: Istanbul Gelisim Universitesi, TÜRKIYE

## Abstract

The purposes of this paper are to develop measurement scale of value co-creation among spectators in professional spectating sporting events by means of Study I and to examine the effects of social media strategies on value co-creation among spectators through Study II. In Study I, a five-phase framework for developing a measurement scale is adopted including items generation, items refinement and edition, exploration of the latent factor structure of the scale and confirmation of reliability, examination of the structure of the factors, and scale validation. In Study II, four quasi-experiments are conducted to investigate the effects of electronic word of mouth, second screen, social media influencer promotion and online donation on value co-creation among spectators. The sample size for Study I and Study II is 830 and 993, respectively. Results obtained from Study I reveal three dimensions of value co-creation among spectators in the context of professional spectating sporting events: event atmosphere, word of mouth, and spectator interaction. Findings from Analysis of Covariance (ANCOVA) in Study II indicate that in Experiment I, positive electronic word of mouth does not help improve value co-creation among spectators while negative electronic word of mouth does decrease value co-creation among spectators. In Experiment II, the second screen under the condition of either positive or negative electronic word of mouth does not exert an influence on value co-creation among spectators. In Experiment III, the effect of social media influencer promotion on value co-creation among spectators is partially supported. In Experiment IV, under the condition of positive electronic word of mouth, the group of donation reveals greater mean score of event atmosphere than that of the counterparts for the group without donation. The findings of this study not only enrich sport management literature in terms of value co-creation, but also provide empirical evidence and practical implications for decision makers of professional spectating sporting events in terms of social media strategies.

## Introduction

Value cocreation refers to the process through which customers provide ideas to organizations, shape organizational processes, and influence other customers [1]. This concept is relevant in the context of the sports industry, where spectators and fans not only engage with sports but also share their opinions about events, brands, and services within communities of like-minded individuals [2]. This interaction affects sports organizations and other spectators, leading spectators to assume the roles of coproducers and cocreators of value in new services [3]. Consumers often derive enjoyment from and feel passionate about spectator sports, supporting the idea that consumers can cocreate value for others [4]. Although extensive research has been conducted on value cocreation, few studies have focused on customer-to-customer value cocreation, with even fewer considering spectators at sports events [5,6].

Insufficient research has explored how value cocreation can be measured in the field of sports event marketing [7]. Although several empirical studies have proposed multidimensional and hierarchical scales for this purpose [8–11], these scales have not been specifically designed for the context of sports event marketing, which limits their ability to measure value cocreation among spectators. For example, one study focused only on helping in the context of value cocreation, which deviates from the goal of developing comprehensive measurement tools and conflicts with existing scale structures [6]. To address this gap in the literature, the present study developed a scale specifically for measuring value cocreation among spectators in the context of sports event marketing, providing a valid and reliable tool that can be used in future research.

The rapid advancement of information technology has extensively influenced business landscapes, including consumer value cocreation [12]. Digital transformation involves radical economic and social changes driven by information technology [13]. Changes related to social media exemplify such transformation because the hyperconnectivity of social media allows for instantaneous communication among users [14]. Sports events also have unique characteristics, such as spectators viewing themselves as experts, which encourages them to share opinions for psychological benefits [15]. The hyperconnectivity of the modern world resulting from the ubiquitousness of social media and the unique characteristics of sports events renders current research on value cocreation less applicable to the modern world and the sports event context. Therefore, further research is required to explore value cocreation among spectators in the contexts of social media and sporting events.

User-generated content is a key feature of social media and is often used to share information [16,17]. User-generated content includes electronic word of mouth, which is a key success factor in e-commerce [18]. Using a second screen has been reported to increase an individual’s learning capability [19,20]. In addition, social media influencers (SMIs) have become increasingly popular, and therefore, social media influencer endorsement is a critical strategy in business communication [21–23]. Another strategy used on social media involves donations, with audiences sponsoring social media influencers. Instagram users pay more attention to brand tags in photos [24]. Research indicates that sports fans often share their opinions on social media [25]. These findings have led to increased academic and professional interest in social media strategies. However, the relationship between these strategies and value cocreation among spectators of sports events remains an underexplored area.

This research offers two key contributions to the literature regarding value cocreation. First, it developed a measurement scale for value cocreation among spectators at professional sports events. Second, the findings from four experiments offer valuable insights that can assist sports event organizers in using social media to enhance value cocreation among spectators at these events.

## Literature review

### Customer-to-customer value cocreation

Value cocreation is commonly defined as a process through which businesses and customers interact to create value [26]. Numerous studies have confirmed that such interaction is crucial [27,28]. However, value cocreation also occurs among customers. When companies are not perceived to be the sole creators of value, the process is determined by the environment and participants involved [29]. Customers who make similar purchases often share interests and motivations, and this influences their service experience and value creation [6]. Therefore, customer-to-customer interaction is a potential factor in value cocreation [4]. However, few studies have explored customer-to-customer value cocreation [5], which involves benefits gained through resource integration during customer interactions [30]. Value cocreation constitutes a distinct social structure and shared image [28]. Value cocreation involves customers providing ideas to organizations, shaping organizational processes, and influencing other customers [1]. These interactions involving the shared interests and motivations affect service experience and value creation, and therefore, customer interaction is a key factor in value cocreation [6].

Customer-to-customer value cocreation is influenced by various factors and social attitudes in addition to potential social interactions [31]. When individuals are more aware of others and engage in longer interactions, value cocreation becomes more complex. User experience sharing may explain value cocreation behavior; it allows companies and customers to benefit from resource integration and interactivity through, for example, social media platforms [32]. A study reported that customer interactions on social media lead to value cocreation primarily through information sharing. Therefore, high levels of value cocreation are likely to occur in social media environments [33]. Although the importance of value cocreation has been recognized in the literature, its role in sports contexts remains underexplored.

### Theoretical background of value cocreation in the context of sports events

Service-dominant logic suggests that both producers and consumers are involved in the process of value creation [3]. Therefore, value cocreation is not solely the responsibility of a firm; consumers also play a role [34]. In the sports industry, value cocreation involves activities related to teams, leagues, and media entities [35]. Sports organizations cocreate value with consumers rather than independently creating value [6,36]. Both sports service providers and consumers offer value propositions that can be integrated into the sports community through the sharing of various resources, such as knowledge, skills, and technology [37]. A sports management study examined interactions between club employees and consumers from a service-dominant logic perspective [38].

Value cocreation is a well-studied phenomenon; however, few studies have focused on customer-to-customer value cocreation in a sports context [5,6]. Spectator sports events are hedonic services, and spectator enthusiasm is a key feature of the events [39]. Sports events also have other unique features, including competition and cooperation among organizations, consumers acting as experts, fluctuating demand, intangible products, simultaneous production and consumption, social facilitation, product inconsistency, product uncontrollability, reliance on ancillary products, and involving both consumer and industrial products [40]. These features suggest that interactions among spectators on social media platforms can attract new participants. As a result, exploring spectator-to-spectator value cocreation in sports is theoretically justified.

Value cocreators may be individual customers with partners (e.g., spouses), known groups (e.g., friends), temporarily unknown groups (e.g., spectators at a sports event), or persistent groups (e.g., loyal and organized members of sports event spectator groups) [41]. In the present study, spectator-to-spectator value cocreation in sports event marketing is defined as the process of creating value through interactions among these different types of spectator groups.

### Value cocreation scales

Existing scales for measuring value cocreation focus on such cocreation between businesses and customers, not that among customers [8–11]. Therefore, these scales are inadequate for measuring spectator value cocreation in sports events. This mismatch was evident in a study that measured value cocreation by considering word-of-mouth behavior and helping as customer citizenship behavior [6]. Thus, the main focus of the present study was to develop a scale specifically for measuring spectator-to-spectator value cocreation in sports events to address a gap in the literature.

### Formation of hypotheses

#### Effect of type of electronic word of mouth on spectator-to-spectator value cocreation.

Marketing strategies that include the use of user-generated content predict consumer word of mouth and value cocreation [18]. User-generated content is conceptually similar to value cocreation [42]. Empirical studies have indicated that consumers are more likely to engage in value cocreation behavior after being exposed to positive electronic word of mouth than after being exposed to negative electronic word of mouth [43,44]. Additionally, sports fans often share their opinions on social media [25]. Accordingly, the following hypotheses were proposed.

H1-1: Positive electronic word of mouth promotes spectator-to-spectator value cocreation.

H1-2: Negative electronic word of mouth discourages spectator-to-spectator value cocreation.

#### Effect of second screen on spectator-to-spectator value cocreation.

Research has indicated that usage of a second screen leads to better learning outcomes [19, 20] and is linked to higher levels of engagement [45]. An example of engagement in a sports context is supporting one’s favorite team [46]. Spectators using a second screen tend to share and recommend ideas on social media [47]. As such, the following hypothesis was proposed.

H2: Under conditions of positive electronic word of mouth, usage of a second screen predicts a greater likelihood of engaging in spectator-to-spectator value cocreation behavior than does usage of only a first screen.

#### Effect of social media influencers on spectator-to-spectator value cocreation.

Strategies involving social media influencers have become increasingly popular in business marketing [21–23]. According to the stimulus–organism–response framework, social media influencers encourage individuals to pay more attention to brands and even make brand recommendations [48]. Social media influencers promote engagement among consumers by using strategies that involve repetitive electronic word of mouth [49]. Hence, the following hypothesis was proposed.

H3: Under the condition of positive electronic word of mouth, social media influencer encourage spectator-to-spectator value cocreation behavior.

#### Effect of online donations on spectator-to-spectator value cocreation.

Monetary donations can be made through online platforms. Instagram users are often more likely to purchase from brands showcased in Instagram posts [24]. On YouTube, donations made during livestreams are presented during live chats in an eye-catching manner. As a consequence, under conditions of positive electronic word of mouth, participants receiving online donation stimuli exhibit greater spectator-to-spectator value cocreation behavior. Accordingly, the following hypothesis was proposed.

H4: Under the condition of positive electronic word of mouth, online donations encourage spectator-to-spectator value cocreation behavior.

## Materials and methods

### Study I: Development of spectator-to-spectator value cocreation scale

This study adopted an established framework to develop an effective measurement scale for spectator-to-spectator value cocreation in sports events[50,51]. The study performed item analysis, purification and refinement of scale items, exploration of the scale’s latent factor structure and confirmation of reliability, generalization of factor structure, and validation of the scale.

#### Phase I: Item generation.

A questionnaire was developed to obtain information about the factors that influence value cocreation at sports events. The questionnaire items were open-ended. For example, one of the items was, “What factors do you think are crucial in spectator-to-spectator value cocreation?” Responses were categorized, and factors that were reported by at least three respondents were considered critical factors. Three sports management scholars were invited to review the critical factors to confirm their content validity.

#### Phase II: Purification and refinement of scale items.

Item analysis was conducted in this phase. Items with correlation coefficients of less than 0.3 were deleted.

#### Phase III: Exploration of latent factor structure and confirmation of reliability.

Exploratory factor analysis was conducted to explore the underlying structure of the collected data. The principal component method was employed for factor extraction. Promax rotation was applied to the extracted factors. The number of factors to be extracted was determined using the criterion of eigenvalues being greater than one. Items with cross-loadings on multiple factors (i.e., those with factor loadings exceeding 0.4 on more than two factors) were removed [52].

#### Phase IV: Generalization of factor structure.

Confirmatory factor analysis was performed to verify the factor structure obtained in the previous phase. According to the recommendations of a previous study [52], a measurement model with construct validity should meet the following conditions: first, the standardized factor loadings should be at least 0.5, indicating convergent validity; second, the individual average variance extracted for each factor should be greater than the square of the correlation between factors, indicating discriminant validity. The reliability of the scale dimensions was assessed in terms of composite reliability. Composite reliability values of more than 0.7 were considered acceptable.

#### Phase V: Validation and verification of the scale.

Confirmatory factor analysis was performed again to verify the factor structure obtained in the fourth phase. The stability of the factor structure and the applicability of external samples were examined.

### Study II: Measurement of factors affecting spectator-to-spectator value cocreation

#### Experiment 1.

Positive, negative, and control electronic word of mouth groups were established. Stimuli were presented in the manner of a livestreaming layout. After being recruited, participants were serially numbered. The serial number was then randomly divided into three groups, namely positive, negative, and control groups. Participants who were randomly assigned to the positive electronic word of mouth group received positive electronic word of mouth information related to the franchise. according to the serial number whereas those who were randomly assigned to the negative electronic word of mouth group received negative information related to the franchise according to the serial number. The control group was presented with health information that was irrelevant to electronic word of mouth. A manipulation check item on the type of electronic word of mouth was adopted from [53]. The item was “For me, this message regarding the New Taipei Kings is…” This item was rated on a 7-point differential semantic scale ranging from positive to negative. An independent-samples t test revealed a significant difference in the ratings for positive and negative word of mouth (*M*_*Positive eWOM*_ = 5.21, *SD*_*Positive eWOM*_ = 1.52, *M*_*Negative eWOM*_ = 3.97, *SD*_*Negative eWOM*_ = 1.80, *t* = 5.69, *p* <.01), suggesting that the manipulation of the type of electronic word of mouth was effective.

The participants were given a link to a positive-, negative-, or control-group version of the online survey and were then presented with an option to consent to participation in this research. They answered questions regarding their identification with the New Taipei Kings basketball team. After being shown positive, negative, or control group electronic word of mouth stimuli, they responded to a manipulation check item and then completed the spectator-to-spectator value cocreation scale.

#### Experiment 2.

After being recruited, participants were serially numbered. The serial number was then randomly divided into two groups. One group, namely with second screen and without second screen. Participants were randomly divided into second screen and no second screen groups according to the serial number. Both groups watched a basketball livestreaming event. The second screen group that had a second screen had a second screen in the lower-right corner of the livestream which presented positive electronic word of mouth content. The procedure was similar to that in Experiment 1.

#### Experiment 3.

After being recruited, participants were serially numbered. The serial number was then randomly divided into two groups, namely with and without social media influencer promotion. Participants were randomly divided into social media influencer promotion and no social media influencer promotion groups according to the serial number. Both groups watched a basketball livestreaming event. The social media influencer promotion group was additionally joined by a social media influencer, who promoted the event. The procedures were applied in the manner similar to that in Experiment 1.

#### Experiment 4.

After being recruited, participants were serially numbered. The serial number was then randomly split into two groups, namely with and without online donation. Participants were randomly divided into online donation and no online donation groups according to the serial number. Both groups watched a basketball livestreaming event. The online donation group additionally had online donation on the right side of the screen which appears positive electronic word of mouth. The procedures were applied in a manner similar to that in Experiment 1.

### Research protocol and participants

This research was approved by the Ethics Committee of National Taiwan University (protocol code 202112ES054, approval date 20220121). The recruited participants were briefed on the study and asked to provide their written consent. The participants in Study I were individuals who had watched the Taipei Fubon Braves in Taiwan Professional Basketball League (P. LEAGUE+) games during the 2022–2023 Championship season. A total of 830 participants were recruited between February and March 2023. Participants in Study II were individuals aged 20 years or older who had attended home games of the Taipei Fubon Braves team. Sampling was conducted using quota sampling on the basis of seating sections. One half of participants were from lower seats while the other half of participants were from upper seats. For Study II, the target population was fans of the New Taipei Kings. Recruitment was conducted in June 2023, with a total of 993 participants recruited through the team’s official social media fan page.

### Data analysis

Data were analyzed using descriptive statistics, exploratory factor analysis, confirmatory factor analysis, and one-way analysis of covariance (ANCOVA). Data were analyzed in SPSS 18.0 and LISERL 8.80. The reason why analysis of covariance was conducted was that analysis of covariance can increase statistical power, which can reduce Type II error rate[54]. In addition, statistically adjusting for the effects of covariates on dependent variable can help account for group difference[54]. Team identification is associated with consumers’ psychological constructs in the sports context and has a significant impact on perceptions, attitudes, and consumption behaviors[55]. In this study, team identification was included as a covariate to eliminate its influence on the constructs under study, reduce bias on the dependent variables, and enhance statistical power.

## Results

### Study I: Development of spectator-to-spectator value cocreation scale

A spectator-to-spectator value cocreation scale was developed, and the validity and reliability of the scale was verified. The scale had three dimensions: event atmosphere, word of mouth, and spectator interaction (Table 1). Confirmatory factor analysis revealed that the scale had satisfactory psychometric properties, including convergent validity and discriminant validity (Table 2 and Table 3). A second confirmatory factor analysis revealed similar outcomes.

**Table 1:** Exploratory factor analysis (N = 130) results for spectator value cocreation in sports events (N_1_ = 300, N_2_ = 400).

Factor/Item	*M*	*SD*	*FL*
Event Atmosphere, [α =.89(.92)]			
1. I believe that the players’ performance during the game increases the interaction among spectators.	6.19 (6.08)	1.13 (1.32)	.966
2. The matchup between teams increases interaction among spectators.	6.20 (6.09)	1.15 (1.32)	.851
3. The team’s performance enhances interaction among spectators.	6.14 (5.98)	1.15 (1.33)	.841
4. The atmosphere of the home games increases the spectators’ viewing experience.	6.36 (6.18)	1.06 (1.29)	.827
5. The preference or identification with a particular star player elevates interaction and discussion among spectators.	6.32 (6.23)	1.02 (1.26)	.759
Word of mouth, [α =.94(.92)]			
1. Based on my viewing experiences, I would recommend P.LEAGUE+ games featuring the Fubon Braves to others.	6.00 (5.82)	1.34 (1.54)	.943
2. Based on my viewing experiences, I would recommend P.LEAGUE+ games featuring the Fubon Braves to my family.	5.78 (5.71)	1.51 (1.59)	.942
3. Based on my viewing experiences, I would recommend P.LEAGUE+ games featuring the Fubon Braves to people of my age.	5.97 (5.84)	1.33 (1.48)	.914
4. Based on my viewing experiences, I would recommend P.LEAGUE+ games featuring the Fubon Braves to those interested in basketball.	6.00 (5.97)	1.34 (1.37)	.898
Spectator interaction, [α =.79(.87)]			
1. I feel satisfied with being part of my friends or family at the game.	5.80 (5.65)	1.36 (1.54)	.952
2. Through interacting and discussing game content with other spectators, viewing pleasure is enhanced.	6.14 (5.89)	1.12 (1.42)	.840
3. I feel great about the passion among spectators at the game.	5.66 (5.49)	1.40 (1.54)	.743

Numbers in parentheses refer to the *N*_*2*_ sample. *N*_*1*_ = First analysis sample; *N*_*2*_ = Second analysis sample. α = Reliability. KMO and Bartlett test (*χ*^2^ = 1376.61, *df* = 66, *p* <.01). FL: Factor loading for exploratory factor analysis *p <.05.

**Table 2:** Confirmatory factor analysis results for spectator value cocreation in sports events (N_1_ = 300, N_2_ = 400).

Factor/Item	*λ*	*T*
Event Atmosphere, [AVE=.66(.69)]		
1. I believe that the players’ performance during the game increases the interaction among spectators.	.76 (.81)	–
2. The matchup between teams increases interaction among spectators.	.77 (.83)	13.51 (19.23)
3. The team’s performance enhances interaction among spectators.	.81 (.81)	14.29 (18.52)
4. The atmosphere of the home games increases the spectators’ viewing experience.	.82 (.86)	14.61 (20.22)
5. The preference or identification with a particular star player elevates interaction and discussion among spectators.	.78 (.84)	13.67 (19.28)
Word of mouth, [AVE=.77(.74)]		
1. Based on my viewing experiences, I would recommend P.LEAGUE+ games featuring the Fubon Braves to others.	.65 (.85)	–
2. Based on my viewing experiences, I would recommend P.LEAGUE+ games featuring the Fubon Braves to my family.	.87 (.87)	19.96 (22.63)
3. Based on my viewing experiences, I would recommend P.LEAGUE+ games featuring the Fubon Braves to people of my age.	.85 (.89)	22.53 (23.35)
4. Based on my viewing experiences, I would recommend P.LEAGUE+ games featuring the Fubon Braves to those interested in basketball.	.91 (.84)	22.48 (21.20)
Spectator interaction, [AVE=.57(.70)]		
1. I feel satisfied with being part of my friends or family at the game.	.87 (.87)	–
2. Through interacting and discussing game content with other spectators, viewing pleasure is enhanced.	.70 (.80)	11.68 (18.91)
3. I feel great about the passion among spectators at the game.	.77 (.83)	12.88 (19.97)

Numbers in parentheses refer to the *N*_*2*_ sample. *N*_*1*_ = First analysis sample; *N*_*2*_ = Second analysis sample. CFA = Confirmatory factor analysis; AVE = Average variance extracted; *λ* = Standardized factor loading for CFA; *t* = t value. χ*²/df* = 132.925 (219.273)/51 (51) = 2.61 (4.30); RMSEA =.07 (.09); NFI =.95 (.95); NNFI =.96 (.95); CFI =.97 (.96); GFI =.93 (.91); SRMR =.05 (.03).

**Table 3:** Discriminant validity analysis (N_1_ = 300, N_2_ = 400).

Factor	Event atmosphere	Word of mouth	Spectator interaction
Event atmosphere	**.62(.69)**		
Word of mouth	.42(.64)	**.78(.75)**	
Spectator interaction	.57(.62)	.41(.50)	**.58(.70)**

Diagonal values are the average variance extracted. Semidiagonal values are the square of the construct correlation coefficients.

### Study II: Assessment of social media strategies for spectator-to-spectator value cocreation

#### Experiment 1: Effects of electronic word of mouth on spectator-to-spectator value cocreation.

Experiment 1 examined the effect of type of electronic word of mouth on spectator-to-spectator value cocreation. To elaborate, positive electronic word of mouth promotes spectator-to-spectator value cocreation (H1-1) while negative electronic word of mouth discourages spectator-to-spectator value cocreation (H1-2). The overall model for the event atmosphere dimension from one-way analysis of covariance was statistically significant (*F* = 72.038, *p* <.01). The effect of electronic word of mouth (positive/negative/neutral) on event atmosphere was statistically significant (*F* = 4.01, *p* =.019). No significant difference in the mean scores of event atmosphere was observed between neutral (*M* = 5.56, *SD* = 1.21) and positive (*M* = 5.56, *SD* = 1.20, *p* =.45) electronic word of mouth groups. Therefore, H1-1 was not supported. However, a significant difference in the mean scores of event atmosphere was revealed between neutral (*M* = 5.56, *SD* = 1.21) and negative (*M* = 5.19, *SD* = 1.13, *p* =.006) electronic word of mouth groups. Accordingly, H1-2 was supported.

Likewise, the one-way analysis of covariance for the word-of-mouth dimension indicated that the overall model was statistically significant (*F* = 137.45, *p* <.01). The effect of electronic word of mouth (positive/negative/neutral) on word of mouth was statistically significant (*F* = 4.80, *p* =.009). No significant difference in mean scores of word-of-mouth was found between neutral (*M* = 5.49, *SD* = 1.55) and positive (*M* = 5.55, *SD* = 1.34, *p* =.86) electronic word of mouth groups. Thus, H1-1 was not supported. Nevertheless, a significant difference in the mean scores of word-of-mouth dimension was detected between neutral electronic word of mouth group (*M* = 5.49, *SD* = 1.55) and negative electronic word of mouth group (*M* = 5.09, *SD* = 1.26, *p* =.03). As a result, H1-2 was supported.

Similarly, the overall model of one-way analysis of covariance for the spectator interaction dimension was statistically significant (*F* = 101.64, *p* <.01). The effect of electronic word of mouth (positive/negative/neutral) on spectator interaction was statistically significant (*F* = 2.46, *p* =.09). No significant difference in the mean scores of spectator interaction was indicated between neutral (*M* = 5.59, *SD* = 1.46) and positive (*M* = 5.60, *SD* = 1.33, *p* =.65) electronic word of mouth groups. Therefore, H1-1 was not supported. However, a significant difference in the mean scores of spectator interaction was suggested between neutral electronic word of mouth group (*M* = 5.59, *SD* = 1.46) and negative electronic word of mouth group (*M* = 5.26, *SD* = 1.26, *p* =.006). Consequently, H1-2 was supported.

Overall, the findings from Experiment 1 suggest that positive electronic word of mouth does not improve spectator-to-spectator value cocreation and that negative electronic word of mouth reduces spectator-to-spectator value cocreation.

#### Experiment 2: Effects of second screen on spectator-to-spectator value cocreation.

H2 predicted that usage of a second screen demonstrates a greater likelihood of engaging in spectator-to-spectator value cocreation behavior than does without usage of a second screen under conditions of positive electronic word of mouth. Although the overall model of one-way analysis of covariance for the event atmosphere dimension was statistically significant (*F* = 85.46, *p* <.01), the effect of the second screen (yes/no) on event atmosphere was not statistically significant (*F* = 0.77, *p* =.38). Under conditions of positive electronic word of mouth, no significant difference was identified in mean scores of event atmosphere between the usage of second screen group (*M* = 5.47, *SD* = 1.20) and the group without usage of second screen (*M* = 5.55, *SD* = 1.21).

Similarly, while the overall model of one-way analysis of covariance for the word of mouth dimension was statistically significant (*F* = 120.71, *p* <.01), the effect of the second screen (yes/no) on word of mouth was not statistically significant (*F* = 1.14, *p* =.29). Under the condition of positive electronic word of mouth, no significant difference was observed in the mean scores of word of mouth between the usage of second screen group (*M* = 5.50, *SD* = 1.28) and the group without usage of second screen (*M* = 5.59, *SD* = 1.34).

Likewise, despite the statistical significance of overall model of the one-way analysis of covariance for the spectator interaction dimension being revealed (*F* = 108.09, *p* <.01), the effect of the second screen (yes/no) on spectator interaction was not statistically significant (*F* = 1.01, *p* =.32). Under the condition of positive electronic word of mouth, no significant difference was discovered in the mean scores of spectator interaction dimension between the usage of second screen group (*M* = 5.45, *SD* = 1.26) and the group without usage of second screen (*M* = 5.55, *SD* = 1.33) groups. In summary, under the condition of positive electronic word of mouth, the second screen did not influence spectator-to-spectator value cocreation. Therefore, H2 was not supported.

#### Experiment 3: Effect of social media influencers on spectator-to-spectator value cocreation.

H3 investigated whether social media influencer encourages spectator-to-spectator value cocreation behavior under the condition of positive electronic word of mouth. The overall model of one-way analysis of covariance of the event atmosphere dimension was statistically significant (*F* = 47.38, *p* <.01). To illustrate, under conditions of positive electronic word of mouth, the social media influencer promotion group had a greater mean score for event atmosphere (*M* = 5.68, *SD* = 1.14) than did the group without social media influencer promotion (*M* = 5.19, *SD* = 1.13, *F* = 3.05, *p* =.08). Additionally, the overall model of one-way analysis of covariance for the word of mouth dimension was statistically significant (*F* = 81.23, *p* <.01). To elaborate, under conditions of positive electronic word of mouth, the mean score of event atmosphere for social media influencer promotion group (*M* = 5.66, *SD* = 1.16) was greater than that for the group without social media influencer promotion (*M* = 5.09, *SD* = 1.26, *F* = 3.12, *p* =.08). However, no significant difference was identified in the mean scores for spectator interaction (*F* = 2.32, *p* =.13) between the social media influencer promotion group (*M* = 5.77, *SD* = 1.21) and the group without social media influencer promotion (*M* = 5.26, *SD* = 1.26). Overall, the findings suggested that H3 was partially supported.

#### Experiment 4: Effect of online donation on spectator-to-spectator value cocreation.

H4 explored whether online donations encourage spectator-to-spectator value cocreation behavior under the condition of positive electronic word of mouth. The overall model of one-way analysis of covariance for the event atmosphere dimension was statistically significant (*F* = 46.49, *p* <.01). To illustrate, under the condition of positive electronic word of mouth, the mean scores of event atmosphere for online donation group was greater (*M* = 6.05, *SD* = 1.25) than that for group without online donation group (*M* = 5.19, *SD* = 1.13, *F* = 5.55, *p* =.019). In contrast, no significant difference was identified in mean scores of word of mouth (*F* = 1.24, *p* =.27) between the online donation group (*M* = 5.98, *SD* = 1.41) and the group without online donation (*M* = 5.10, *SD* = 1.26). Likewise, no significant difference was detected in the mean scores of spectator interaction (*F* = 1.34, *p* =.25) between the online donation group (*M* = 6.00, *SD* = 1.33) and the group without online donation (*M* = 5.26, *SD* = 1.26). Overall, these findings indicate that H4 was partially supported.

## Discussion

### Study I: Development of spectator-to-spectator value cocreation scale

From the results of Study I, spectator-to-spectator value cocreation scale consists of three dimensions; event atmosphere, word of mouth, and spectator interaction. The first dimension, event atmosphere, was the most notable feature in this study as it differs from factors proposed in other studies on value cocreation. In other words, event atmosphere has not been proposed in the literature on value cocreation but rather exhibits a unique characteristic within the context of spectator sports. Such a finding echoes that atmosphere in sports environments is a significant factor in explaining fan behavior. Empirical studies have reported on the influence of physical, social, and environmental factors in creating atmospheres [56–58]. In commercial settings, the presence of other customers has the potential to exert a strong influence on atmosphere and to make the atmosphere relevant to other customers [59]. Similarly, a study [60] reported that fans’ experiences with respect to sports venue environments can influence their willingness to participate, suggesting that atmosphere may be a key factor in spectator-to-spectator value cocreation. Therefore, this study posits that event atmosphere is an irreplaceable factor in value cocreation in spectator sports events.

The second dimension was word of mouth. This finding aligns with that of another study [6]. One of the shared characteristics among sports fans is their tendency to spread word of mouth through social media networks [25]. Sports events involve various elements, such as live activities, cheerleading squads, online promotions, and the excitement of the event itself. Enthusiasm is a common characteristic among sports event spectators [39]. The aforementioned elements may stimulate excitement among spectators [59]. Additionally, individuals tend to trust information from recommenders; they often make purchase decisions on the basis of recommendations, leading to a reward for successful recommendations made to relatives, friends, and family [61]. A previous study [62] reported that potential customers are more likely to be influenced by word of mouth from existing customers when they perceive themselves to be similar to those existing customers. In a sports context, this influence can affect customers’ willingness to attend future sports events. Research [63] on value creation in the context of live sports events revealed that environmental elements, such as venue aesthetics and seating comfort, and core services, such as player skills and team performance, are highly valued by spectators. Creating social experiences can prompt spectators to share team or sports-related information through word of mouth recommendations on social media platforms. Spectators may also engage in extended discussions on social media platforms or elsewhere after an event, including exchanging opinions on the event process, team performance, and other factors. These discussions are crucial for fans. This finding echoes previous findings [6] indicating that feelings induced by other spectators may lead to behaviors related to word of mouth.

Spectator-to-spectator value cocreation is unique in that it involves spectator interaction, which includes communication with the environment and people. Service providers should be aware of the importance of social interaction to spectators and should provide a platform that facilitates communication and interaction among athletes, fans, sports brands, and sports organizations. This would enable spectators to connect with each other and cocreate event experiences [64]. Research [65] suggests that value cocreation can be indirectly viewed as interaction, where customers support brands through rich experiences and reinforce identification with the brand through other customers. This encourages customer identification and builds cohesion among customers. The characteristics of sports event spectators may encourage new spectators, such as spouses and friends, to interact on communication platforms [41], where sports event spectators may discuss the conditions of a game. Spectators who are attending a game for the first time may be unfamiliar with certain aspects of the event. However, spectators with experience may introduce or explain the event to these new spectators. In addition, those who hold season tickets may feel a stronger loyalty to the team, players, and event, positively reinforcing related behaviors.

### Study II: Four quasi-experiments on spectator-to-spectator value cocreation

The findings from Experiment 1 were that positive electronic word of mouth did not improve spectator-to-spectator value cocreation. However, negative electronic word of mouth reduced spectator-to-spectator value cocreation. Overall, the results of Experiment I are consistent with those reported in the literature, indicating that consumers who value cocreation behavior tend to be influenced by electronic word of mouth [43,44]. The finding that positive electronic word of mouth does not enhance value cocreation may be partially explained by confirmation bias. Confirmation bias refers to the tendency of individuals to favor information that confirms their beliefs [66]. In line with confirmation bias, the reason value cocreation is not elevated by positive electronic word of mouth can be inferred from sports fans’ often strong psychological attachment to a particular sports franchise. This may, in turn, explain the finding of positive electronic word of mouth being less effective in boosting spectator-to-spectator value cocreation.

The results from Experiment II indicate that a second screen did not influence spectator-to-spectator value cocreation. This finding contradicts that of a study that demonstrating that sport fans with a second screen tend to make recommendations on social media [47]. Methodological differences may help explain the different results. Specifically, the current research adopted an experimental design approach. Other studies have been correlational and have used structural equation modeling. Further research may be required to confirm this finding.

Experiment III partially supported H3. Under conditions of positive electronic word of mouth, social media influencer promotion increased the mean score for the event atmosphere dimension. This finding aligns with those of other studies that have demonstrated that repetitive promotion by social media influencers influences consumers’ engagement on social media and recommendation of brands [48,49]. The findings of this study can be explained by the concept of the parasocial relationship. Parasocial relationships occur when after viewing their content on social media, audiences perceive social media influencers as friends, leading to trust on and reliance in the influencer [67].

Experiment IV partially supported H4. Under conditions of positive electronic word of mouth, online donations resulted in a higher mean score for the event atmosphere dimension. This finding is consistent with that of a study demonstrating that Instagram users pay salient attention to brand tags presented in the form of photograph [24]. The finding that online donations are often presented in the form of a photograph and placed in a position that can be easily seen aligns with the theory of prosocial behavior. Prosocial behavior refers to actions that conform to social norms and benefit individuals, groups, or society [68].

### Implications

This study has several managerial and academic implications. The scale for measuring spectator-to-spectator value cocreation can be used in several areas of sports management practice to assess strategies for increasing spectator-to-spectator value cocreation. First, sports organizations can use this scale to survey and understand the tendency to engage in spectator-to-spectator value cocreation at sports events to gain insights and maximize behaviors conducive to spectator-to-spectator value cocreation. Second, understanding the effects of social media strategies on spectator-to-spectator value cocreation can enable organizations to develop relevant activities and tactics to further encourage value cocreation behavior on social media. Additionally, regularly measuring spectator-to-spectator value cocreation behavior can help managers monitor changes in value cocreation behavior and devise relevant strategies for different periods. Last, the measurement scale developed in and the experimental findings from this research enriches the literature related to value cocreation in sports event management and to social media strategies for sports events.

### Research limitations and directions for future studies

This study has several limitations. First, the study focused on spectator-to-spectator value cocreation only in the P. LEAGUE+ professional basketball league, which may limit its generalizability. Given the diversity of sports events, future research may investigate spectator-to-spectator value cocreation behaviors in different sports, cultural settings, and league structures to enhance the scale’s generalizability. Additionally, this study was a cross-sectional study. A longitudinal study should be conducted to track spectator-to-spectator value cocreation over time. Future research could also expand upon this study by identifying relevant influencing variables, such as whether spectator-to-spectator value cocreation can predict team loyalty.

## Conclusion

This study developed a valid and reliable psychometric measurement scale of spectator-to-spectator value cocreation. The scale considers the characteristics of spectator sports events and comprehensively captures consumer behavior in this context. Four experiments were conducted to examine the effects of social media strategies on spectator-to-spectator value cocreation, and valuable insights were derived from these experiments. Overall, this study not only advances our academic and practical understanding of spectator-to-spectator value cocreation but also enriches the literature regarding social media strategies for spectator-to-spectator value cocreation.

## Supporting information

S1 File Study 1_EFA. (XLSX)

S2 File Study 1_CFA1. (XLSX)

S3 File Study 1_CFA2. (XLSX)

S4 File Study 2_Experiment 1. (XLSX)

S5 File Study 2_Experiment 2. (XLSX)

S6 File Study 2_Experiment 3. (XLSX)

S7 File Study 2_Experiment 4. (XLSX)

S8 File Spectator-to-spectator value cocreation scale. (PDF)
