# Supplementary material for: Value cocreation among spectators in professional spectator sports events: Scale development and effects of social media strategies
Source: PLoS One. 2025 May 23;20(5):e0320927. doi: 10.1371/journal.pone.0320927 (PMC12101675; doi:10.1371/journal.pone.0320927)
Supplement: S8 File — (PDF) [file pone.0320927.s008.pdf]

spectator-to-spectator value cocreation scale

Factor/Item
**Event Atmosphere**
1. I believe that the players' performance during the game increases the interaction among spectators.
2. The matchup between teams increases interaction among spectators.
3. The team's performance enhances interaction among spectators.
4. The atmosphere of the home games increases the spectators' viewing experience.
5. The preference or identification with a particular star player elevates interaction and discussion among spectators.
**Word of mouth**
1. Based on my viewing experiences, I would recommend P.LEAGUE+ games featuring the Fubon Braves to others.
2. Based on my viewing experiences, I would recommend P.LEAGUE+ games featuring the Fubon Braves to my family.
3. Based on my viewing experiences, I would recommend P.LEAGUE+ games featuring the Fubon Braves to people of my age.
4. Based on my viewing experiences, I would recommend P.LEAGUE+ games featuring the Fubon Braves to those interested in basketball.
**Interactive**
1. I feel satisfied with being part of my friends or family at the game.
2. Through interacting and discussing game content with other spectators, viewing pleasure is enhanced.
3. I feel great about the passion among spectators at the game.
